# *CyRSoXS*: a GPU-accelerated virtual instrument for polarized resonant soft X-ray scattering

**DOI:** 10.1107/S1600576723002790

**Published:** 2023-05-23

**Authors:** Kumar Saurabh, Peter J. Dudenas, Eliot Gann, Veronica G. Reynolds, Subhrangsu Mukherjee, Daniel Sunday, Tyler B. Martin, Peter A. Beaucage, Michael L. Chabinyc, Dean M. DeLongchamp, Adarsh Krishnamurthy, Baskar Ganapathysubramanian

**Affiliations:** a Department of Mechanical Engineering, Iowa State University, Ames, IA 50010, USA; b Material Measurement Laboratory, National Institute of Standards and Technology (NIST), Gaithersburg, MD 20899, USA; c Materials Department, University of California, Santa Barbara, CA 93106, USA; d NIST Center for Neutron Research, National Institute of Standards and Technology (NIST), Gaithersburg, MD 20899, USA; Argonne National Laboratory, USA

**Keywords:** CyRSoXS, virtual instruments, polarized resonant soft X-ray scattering, P-RSoXS

## Abstract

Polarized resonant soft X-ray scattering (P-RSoXS) has emerged as a powerful synchrotron-based tool that combines the principles of X-ray scattering and X-ray spectroscopy. P-RSoXS provides unique sensitivity to molecular orientation and chemical heterogeneity in soft materials such as polymers and biomaterials. Quantitative extraction of orientation information from P-RSoXS pattern data is challenging, however, because the scattering processes originate from sample properties that must be represented as energy-dependent three-dimensional tensors with heterogeneities at nanometre to sub-nanometre length scales. This challenge is overcome here by developing an open-source virtual instrument that uses graphical processing units (GPUs) to simulate P-RSoXS patterns from real-space material representations with nanoscale resolution. This computational framework – called *CyRSoXS* (https://github.com/usnistgov/cyrsoxs) – is designed to maximize GPU performance, including algorithms that minimize both communication and memory footprints. The accuracy and robustness of the approach are demonstrated by validating against an extensive set of test cases, which include both analytical solutions and numerical comparisons, demonstrating an acceleration of over three orders of magnitude relative to the current state-of-the-art P-RSoXS simulation software. Such fast simulations open up a variety of applications that were previously computationally unfeasible, including pattern fitting, co-simulation with the physical instrument for *operando* analytics, data exploration and decision support, data creation and integration into machine learning workflows, and utilization in multi-modal data assimilation approaches. Finally, the complexity of the computational framework is abstracted away from the end user by exposing *CyRSoXS* to Python using *Pybind*. This eliminates input/output requirements for large-scale parameter exploration and inverse design, and democratizes usage by enabling seamless integration with a Python ecosystem (https://github.com/usnistgov/nrss) that can include parametric morphology generation, simulation result reduction, comparison with experiment and data fitting approaches.

## Abstract 2

This article presents *CyRSoXS* – an open-source virtual instrument that uses GPUs to simulate polarized resonant soft X-ray scattering (P-RSoXS) patterns from real-space material representations. It is significantly faster than the current state-of-the-art software, and it enables quantitative extraction of orientation information from P-RSoXS data. This enables a wide range of applications, including pattern fitting, co-simulation, data exploration, machine learning workflows and multi-modal data assimilation approaches.

## 1. Introduction

Developing process–structure–property relationships is a central pillar of materials science and engineering research. Understanding the effect of composition, structure and processing on the performance of a material can enable the intelligent and efficient tuning of the process variables to improve the end performance of the material in a given application. With these process–structure–property relationships, the exciting goal of designing new materials instead of discovering them becomes a reality. Thus, there is an ever-present need to develop new characterization methods to elucidate material structure with increasing detail and clarity.

Structural characterization is particularly challenging in soft matter due to its semi-disordered nature. Some important aspects of soft-matter structure include spatial heterogeneities in composition, density, molecular orientation/conformation and degree of order. Recent advances in synthesis and mater­ials processing have unlocked access to systems in which all aspects of soft-matter structure might ultimately be controlled by design. However, despite enormous progress in the capability and speed of characterization methods across many length scales, it remains a fundamental and pervasive challenge to assimilate efficiently, rigorously and robustly the materials structure characterization data streams into a self-consistent *digital twin* that describes the material structure. If achieved, the resultant comprehensive structural description would allow us to understand, predict and eventually control how material properties arise from a complex interplay of different aspects of structure across relevant length scales.

In this context, there have been recent efforts to integrate computational tools with experimental data streams. Virtual instruments that mimic the physical principles of the characterization method – X-ray diffraction, light spectroscopy or electron transmission (Wessels & Jayaraman, 2021 ▸; Mukherjee *et al.*, 2021 ▸; Pryor *et al.*, 2017 ▸; Reynolds *et al.*, 2022 ▸) – can transform how downstream analysis of experimental data streams is performed. For instance, a virtual instrument can enable rapid data quality evaluation and provide statistically rigorous estimates of when enough data have been collected. Such approaches can maximize the utilization of heavily subscribed instruments at centralized facilities such as X-ray and neutron sources. Furthermore, a virtual instrument can allow principled down-selection of plausible hypotheses for developing structure–property relationships. Such virtual tools also allow formal analysis and characterization of uncertainty, identify the most sensitive features, and allow *in silico* experimentation before performing physical experiments, for greater efficiency in experiment execution. Finally, the success of artificial intelligence and machine learning (AI/ML) methods (Axelrod *et al.*, 2022 ▸; Vasudevan *et al.*, 2021 ▸; Guo *et al.*, 2021 ▸; Gomes *et al.*, 2019 ▸) points to the possibility of integrating experimental data with a virtual instrument to provide automated and formal approaches to assimilating complementary data streams – for instance, real space (electron microscopy) and frequency space (X-ray diffraction) – to create a self-consistent and multimodal digital twin.

Polarized resonant soft X-ray scattering (P-RSoXS) is a recently developed technique with unique characterization abilities (Collins & Gann, 2022 ▸) and is an excellent candidate for developing a virtual instrument. Typical scattering experiments performed at hard X-ray energies provide a very low contrast between organic constituents in a material. P-RSoXS overcomes this limitation by combining conventional small-angle X-ray scattering (SAXS) with soft X-ray spectroscopy to yield a tunable scattering contrast. The energies of this soft X-ray beam are scanned across the absorption edges of the light elements (C, N, O) commonly found in organic materials, often yielding significant contrast variation and substantially improved signal-to-noise ratio for organic systems. P-RSoXS thus provides a path to probe the structure in the nanometre to micrometre range with both chemical and physical sensitivity, without the need to perturb the system with labels such as the heavy element ‘stains’ commonly used to enhance SAXS or the radioisotopes commonly used to enhance small-angle neutron scattering (SANS). The contrast enhancement makes P-RSoXS particularly useful for probing the structure of thin (<200 nm) films, samples that are challenging for hard X-rays and neutrons due to the small scattering volumes. Composition contrast with P-RSoXS is so significant that short exposures of thin films (less than 1 min at normal incidence) at resonant energies with high contrast will yield patterns of a quality similar to conventional bulk SANS patterns requiring millimetre-scale sample volumes and hours to collect. The approach also does not require the grazing-incidence geometries which are commonly used to gain signal in the X-ray scattering of thin films.

The variable sensitivity of P-RSoXS to each chemical bond can amplify the scattering intensity even with only small chemical differences between materials, which enables the extraction of useful structure information for heterogeneous materials. A unique aspect of P-RSoXS is that it is sensitive to molecular orientation via interaction of the soft X-ray electric field vector with oriented transition dipoles within the sample. Complex P-RSoXS patterns can arise from orientational heterogeneities. This unique aspect of P-RSoXS provides exciting opportunities for characterizing previously unmeasurable features of the structure of soft materials, but it makes adapting conventional SAXS or SANS analysis approaches nearly impossible because the material properties that affect contrast in those techniques are effectively scalar quantities. A new analysis framework is required to represent independent fluctuations in material composition and molecular orientation on sub-nanometre length scales. The availability of a virtual analog to P-RSoXS would enable the discovery and quantification of structure in complex chemically heterogeneous soft systems. Motivated by this exciting promise, here we describe our development of *CyRSoXS*, a fast graphics processing unit (GPU)-accelerated virtual instrument for P-RSoXS.

To dispel any questions regarding which technique we address herein, we note that, because P-RSoXS is not yet a mainstream technique, a variety of different acronyms have been proposed for it, including ‘R-SoXS’ and ‘PAXS’ (Gann *et al.*, 2016 ▸). The community now appears to have settled on ‘RSoXS’ and ‘P-RSoXS.’ It is not uncommon for practitioners to use only ‘RSoXS’ when exploiting its composition contrast capabilities and to use ‘P-RSoXS’ when adding its orientation contrast capabilities. We should mention, however, that these contrast modes are intrinsically linked. It is not possible to perform RSoXS without polarization and its concomitant molecular orientation sensitivity. Even circular polarization will yield patterns that can be significantly affected by mol­ecular orientation effects. These principles suggest that model-free composition-only analyses of P-RSoXS in systems having significant but ignored molecular orientation fluctuations may yield incorrect results, a situation that could be greatly improved with a fast virtual instrument.

The current state-of-the-art P-RSoXS simulator, developed by Gann *et al.* (2016 ▸) in *IgorPro*,^1^ has been pivotal in answering many scientific questions (Jiao *et al.*, 2017 ▸; Ye *et al.*, 2016 ▸; Song *et al.*, 2018 ▸, 2019 ▸; Mukherjee *et al.*, 2017 ▸; Litofsky *et al.*, 2019 ▸). However, it has limitations on practical deployment in terms of speed and no opportunity for deployment on state-of-the-art high-performance computing clusters. These limitations become most apparent when attempting to fit experimental data using goal-seeking algorithms that adjust material structure input parameters to obtain agreement between simulation and experiment. Such optimization routines require a significant number of forward simulations and thereby motivate the need for a fast forward simulator. The commercial licensing of *IgorPro* further hinders the democratization and availability of the tool to many researchers. There has been rapid growth in interest among materials scientists in using advances in machine learning and data analytics for materials design and exploration. The availability of a fast forward simulator is a critical necessity for data creation and integration into machine learning model operationalization workflows. More practically, since Python has become the *de facto* language for data analysis and machine learning, the currently available simulator does not provide any straightforward integration for researchers to utilize such tools.

As our contribution to this ongoing development, we build upon an earlier framework that modeled the physics of soft X-ray scattering through a heterogeneous thin film (Gann *et al.*, 2016 ▸). In particular, we significantly reduce the execution time and, via integration with a Python ecosystem, incorporate substantial additional functionality. Our key contributions in this paper are as follows:

(i) Accomplishing near real-time simulation of P-RSoXS at sizes/resolutions that were hitherto not possible [up to 2^28^ or 268 million voxels on V100 GPUs; this size is limited purely by the GPU memory (Section 4)].

(ii) Using GPU acceleration to achieve 1000× acceleration over current state-of-the-art approaches. This is achieved by careful design of a ‘GPU-friendly’ data structure and algorithms, including memory and communication considerations.

(iii) Careful software design of a simulation engine that lies at the center of a feature-rich data analysis and model exploration ecosystem when combined with Python code bases for morphology generation, simulation result reduction and data fitting. Python binding democratizes access, simplifies usage and enables seamless integration with AI or ML libraries, and eliminates the bottleneck of input/output (I/O) operation, especially during parameter exploration or inverse design (file I/O is several orders of magnitude slower than memory read/write, so this can quickly become a bottleneck).

(iv) A new and well documented voxel-based material structure data file format in Hierarchical Data Format 5 (HDF5) that includes capabilities for verbose metadata, multiple materials, independent representation of composition and orientation, and an intuitive Euler angle description of material orientation.

(v) An extensive set of validation examples developed by a growing community across multiple institutions.

(vi) Tutorials that serve as unit tests for this open-source framework. The full software stack is open source and requires access to CUDA-enabled hardware.

The rest of the paper is organized as follows: We begin by briefly introducing P-RSoXS in Section 2, followed by a detailed mathematical model in Section 3. We detail the data structures and algorithms in Section 4 and present results including validation cases in Section 5. We show the performance of *CyRSoXS* with varying problem size and scaling to multiple GPUs in Section 6. We discuss integration with Python environments in Section 7, and we conclude by discussing the implications and a path for future development in Section 8.

## 2. Polarized resonant soft X-ray scattering

**Figure 1:**
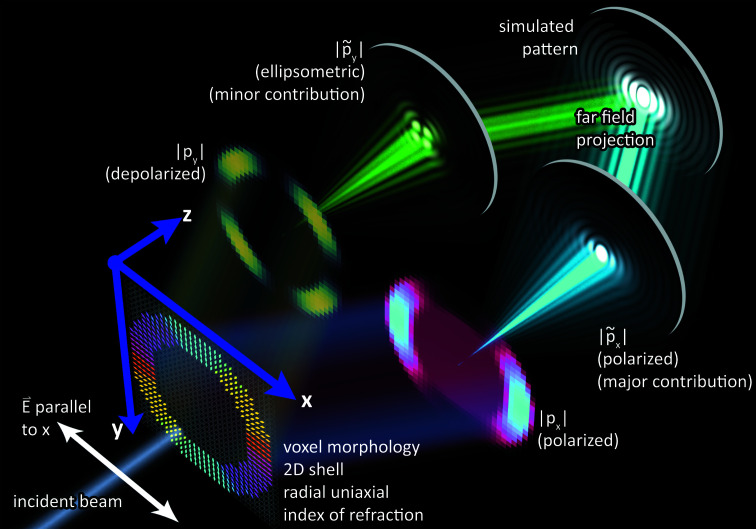
A schematic diagram of P-RSoXS, where a polarized soft X-ray beam passes through a sample, interacting with and scattering off the electrons in that sample; these scattered X-rays are collected on an X-ray sensitive detector.

In P-RSoXS, a polarized soft X-ray beam passes through a sample, interacting with and scattering off the electrons in that sample; these scattered X-rays are collected on an X-ray sensitive detector [typically a charge-coupled device (CCD) or complementary metal–oxide semiconductor (CMOS)]. Fig. 1 ▸ shows a condensed version of the physical principles of P-RSoXS, which are explained in greater detail in a recent comprehensive review of the technique and its application to soft materials (Collins & Gann, 2022 ▸). The strength of the photon–electron interaction depends on the X-ray energy and on the chemistry of the molecules within the sample. At energies far from an absorption edge, X-rays interact equally with all electrons in the sample, and the strength of the interaction scales directly with the electron density. Near an absorption edge, the interaction strength increases dramatically when the incident X-ray energy is commensurate with the energy required to excite an electron resonantly to an unoccupied molecular orbital. The *K* absorption edges of many lightweight elements (C, O, N, F) lie in the soft X-ray energy regime (100 eV ≲ *E*_photon_ ≲ 2 keV); all are commonly exploited in P-RSoXS. Selection of the incident energy near the core binding energy of the electrons makes the technique element specific, whereas the chemical bonds that define the excited-state unoccupied molecular orbital energy make the technique sensitive to specific bonds or chemical groups. The spectroscopic scattering pattern thus provides a tunable chemically sensitive probe of nanoscale and mesoscale components in a heterogeneous complex material (Attwood & Sakdinawat, 2017 ▸).

The resonant soft X-ray absorption is described by a transition dipole moment that couples the initial and final states. The initial state of the electron is a core orbital that is spherically symmetric. Therefore, the geometric dependence of the interaction strength is defined by the unoccupied mol­ecular orbital, which for most soft X-ray resonances can be represented as a vector or plane parallel or perpendicular to the bond (Stöhr, 1992 ▸). Soft X-ray absorption, a principal contributor to scattering contrast, varies as the dot product of the electric field vector and the transition dipole moment. This interaction makes P-RSoXS sensitive to spatial distributions in molecular orientation. For instance, in the case of carbon fused-ring compounds, when the X-ray energy is in resonance with the fundamental carbon electron transition (C1*s* → π*), the molecules exhibit vector transition dipole moments perpendicular to the ring planes (Mannsfeld, 2012 ▸). Two identical molecules oriented differently within a sample will have different interaction strengths with a fixed electric field vector and there will be a scattering contrast between them. If the orientation of these molecules is spatially correlated in a sample, a scattering pattern will be observed. For example, P-RSoXS can detect correlated interfacial molecular orientation regions (such as mixtures of amorphous, semi-crystalline or liquid-crystalline phases). Crystalline, semi-crystalline and liquid-crystalline organic materials have locally large anisotropic bond orientation statistics, impacting the mechanical, optical and electronic properties of these mater­ials. Understanding these relative orientations at different length scales is necessary for a detailed understanding of organic thin-film devices. In addition, P-RSoXS has been shown to reveal local molecular alignment independent of overall crystallinity, and represents an essential new tool for understanding structure–property relationships and examining the connection between transport properties and morphology in organic and hybrid organic–inorganic electronic devices (Mannsfeld, 2012 ▸; Collins & Gann, 2022 ▸; Collins *et al.*, 2012 ▸; Liu *et al.*, 2016 ▸).

The interaction of X-rays with a system can be encoded using a 3D analog of the complex index of refraction, 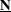 . Each component of this tensor is a function of the dispersive and absorptive components of the index of refraction, *N*_*ij*_(*E*) = *f*(δ(*E*), β(*E*)), where *E* is the photon energy, δ is the dispersive component and β is the absorptive component of the index of refraction. In the hard X-ray regime, far away from the resonance frequency of the constituent atoms, the real part of the complex index of refraction is a scalar proportional to the electron density of the material. The imaginary part is negligible due to low absorption, and the electron-density difference between the constituent materials determines the scattering contrast of the system. However, close to the absorption edge of the constituent atoms, the electrons get excited to the unoccupied molecular states or vacuum and therefore β will naturally exhibit peaks and other absorption features that will differ depending on orientation; changes in δ are also expected due to causality and can be calculated using the Kramers–Kronig relations (Wang *et al.*, 2010 ▸; Watts, 2014 ▸).

## 3. Mathematical model

### 3.1. Notation

The notation used in this paper is as follows.

Vectors are represented as lower-case bold letters, **e**, **p**. Tensors (specifically matrices) are represented as upper-case bold letters with a single underline, 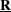 , 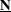 . Scalars are represented as lower-case letters, φ, *n_x_*. The counting (over components) integer is *j*.

Having described the overall mechanism of P-RSoXS, we now detail the mathematics of the simulation.

### 3.2. Morphology

Consider a morphology composed of a *c* component mixture. We discretize the morphology into a uniformly spaced voxel grid. Each voxel contains some (or all) of the *c* components. Each of these components can be either amorphous or oriented. If a component is oriented, we assume that it is well represented by a uniaxial representation.

Note that, while the uniaxial representation is adequate for most use cases currently considered, it has some disadvantages. A key disadvantage is that it will convey less information than other representations. It cannot perfectly represent properties at the molecular level: the simplest representations of mol­ecular-level properties for most molecules would be biaxial. It cannot represent complex orientation distributions at the sub-voxel level: only a single orientation mode is conveyed per material and the ‘shape’ of the distribution is lost.

Notwithstanding the above, a key advantage of the uniaxial assumption is that it is simple and allows the construction of a simple abstract model of properties within a voxel. This abstract model and associated data structures are independent of the material and energy. The abstract model can then be combined with a material library, which can be stored in memory, to allow the same model to be re-used for different materials and different energies. This abstract representation requires only two scalar fractions (volume fraction and orientation fraction) and two Euler angles per material/voxel for the uniaxial assumption. In contrast, a biaxial representation would require five scalar fractions (volume fraction and four orientation mixing parameters) and three Euler angles for a similar abstract model. To represent arbitrary distributions of a biaxial representation in an abstract model would require including non-diagonal elements of the full tensor for 19 unique scalar fractions (volume fraction, and six elements with three coefficients each on the original ‘mol­ecular’ biaxial elements), significantly increasing memory and communication footprints.

A uniaxial representation conveys the necessary properties for materials with a single dominant orientation mode of one particular molecular axis, which we judge will cover a large number of use cases. If a more faithful representation of a multimodal orientation distribution is required, that can be approached in our framework by breaking a component into additional materials with identical dielectric functions and volume fractions that add to the total for that component, but which have distinct orientations reflecting the expected distribution. If a more faithful representation of molecular-level properties is required, it is possible to use a system of uniaxial functions to represent an underlying biaxial function, but that is not a currently supported use case for our approach. We will lay out a clear pathway to relax this assumption and consider biaxial representation in the next release version of *CyRSoXS*.

Each voxel, therefore, has four features associated with each component *j* = 1…*c*:

(i) 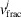 : the fraction of volume occupied by component *j* in this voxel. By definition, 0 ≤ 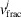 ≤ 1 and the sum 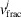 across all *j* (that is, the sum of all volume fractions of all materials) within a voxel is expected to be 1.0. *CyRSoXS* will not check whether they sum to 1.0, although such checking can be done with morphology class methods provided in our broader Python ecosystem. We encourage, as best practice, the use of vacuum as an explicit material in the model, such that model self-consistency is straightforward to confirm.

(ii) *s* ^*j*^: the degree of alignment of component *j* in this voxel. This parameter indicates the volume fraction of component *j* that is oriented (as opposed to unaligned). We expect that 0 ≤ *s* ^*j*^ ≤ 1, but unlike 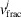 there is no expectation of any constraint involving other materials. *s* ^*j*^ is a relative volume fraction; in other words *s* ^*j*^ is multiplied by 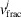 to yield the absolute volume fraction of oriented material *j* in a voxel [see equation (2) below]. This parameter is conceptually identical to the well known uniaxial ‘orientational order parameter’ *S*, but only in the range of 0 ≤ *S* ≤ 1, where *S* = 1 indicates complete alignment with a director (our director is defined by the Euler angles described below) and *S* = 0 indicates an isotropic condition. We note that the orientational order parameter *S* can also include the range −0.5 ≤ *S* ≤ 0, which indicates orientation perpendicular to the director, but we do not support values of *s* ^*j*^ less than zero. Expressing perpendicular orientations should instead be accomplished by explicit adjustment of the Euler angles.

(iii) φ^*j*^: orientation feature 1, defined as the (first) rotation of component *j* about the *z* axis.

(iv) θ^*j*^: orientation feature 2, defined as the (second) rotation of component *j* about the (original) *y* axis.

The last two features represent the Euler angle representation of material orientation in a voxel.

We refrain from providing overly prescriptive guidance on model design because there may be use cases for *CyRSoXS* that we cannot anticipate. However, we offer here some model design choices that have worked well for our internal testing and for many of the validation cases we provide in Section 5. Most models will represent the real-space structure of a thin film, so they will typically have larger *x* and *y* ‘lateral’ dimensions and a smaller *z* ‘height’ dimension. We consider it best practice for *x* and *y* to have the same dimensions and resolutions. Common *x* and *y* dimensions (meaning the length of the whole model on a lateral side) are micrometre scale, perhaps ranging from 0.5 to 5 µm. Common lateral resolutions include 512 × 512, 1024 × 1024 and 2048 × 2048, although larger sizes are possible. The *z* resolution is usually a smaller multiple of 2; common values include 32, 64 and 128. The *z* resolution should be substantially greater than 1 for accurate calculations that involve three-dimensional Ewald sphere components; these are especially important for models that involve significant orientation and pattern anisotropy. The voxels are considered perfect cubes in *CyRSoXS*, such that the model dimensions are the product of the resolution and the length of a voxel side. These model dimensions and resolutions correspond to voxels with side lengths in the range 0.2 to 10 nm. A practical limit on the minimum voxel size could be the diffraction limit of the incident radiation, which for carbon *K*-edge wavelengths is 1.5–2 nm.

These model dimensions and resolutions are compatible with data fusion workflows where real-space images derived from atomic force microscopy, transmission electron microscopy or other imaging methods are used as a foundation for *CyRSoXS* model creation. In some cases such images could be used to assign 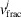 across voxels for different components, depending on the contrast mode of the imaging. The other voxel-level parameters, *s* ^*j*^, φ^*j*^ and θ^*j*^, will most likely not be available from imaging methods because there is a lack of techniques that are sensitive to molecular orientation in soft materials at the nanoscale. (This fact provides much of our motivation for investment in P-RSoXS interpretation.) Hypothesis-driven parametric assignment of *s* ^*j*^, φ^*j*^ and θ^*j*^ might instead be employed. Models built entirely parametrically are certainly possible, as demonstrated for many of our validation cases shown in Section 5.

#### 3.2.1. A brief primer on Euler angles

**Figure 2:**
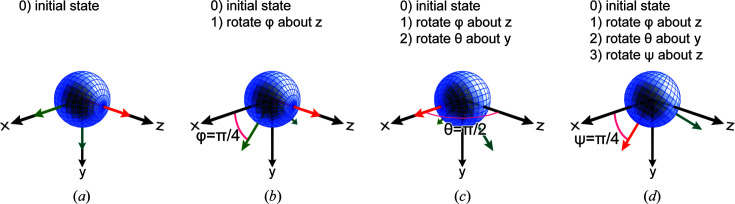
The different steps of the Euler angle rotation. (*a*) Initial state, (*b*) φ around *z*, (*c*) θ around *y* and (*d*) ψ around *z*. The extraordinary optical axis of the uniaxial dielectric function is shown in red; it is initially aligned with the *z* axis. The ordinary optical axes of the uniaxial dielectric function are shown in green; they are initially aligned with the *x* and *y* axes.

For Euler angles, we use the *zyz* convention. We assume that the primary alignment axis starts parallel to the *z* axis (0, 0, 1) [Fig. 2 ▸(*a*)]. This is also the default direction of the simulated incident beam. According to this convention (Fig. 2 ▸), and with reference to the rotation matrices 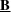 , 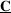 and 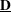 , which are further defined in equation (3) below,

(i) the first rotation is by an angle φ about the *z* axis using rotation matrix 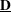 [Fig. 2 ▸(*b*)],

(ii) the second rotation is by an angle θ about the original *y* axis using rotation matrix 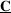 [Fig. 2 ▸(*c*)] and

(iii) the third rotation is by an angle ψ about the original *z* axis using rotation matrix 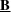 [Fig. 2 ▸(*d*)].

We note that other conventions are possible and have been used in the literature; for instance, Gann *et al.* (2016 ▸) used the vector orientation in 3D space to define an equivalent morphology. These equivalent conventions can be easily transformed into the Euler angles using suitable rotation transformations. A benefit of our convention is its straightforward expandability into a biaxial representation by adding a third Euler angle.

### 3.3. Material properties

As mentioned in Section 2, the interaction of soft X-rays with a material is encoded in the 3D analog, 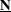 , of the material-specific complex index of refraction. 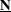 is a 3 × 3 data structure that exhibits energy dependence. For a uniaxial system, 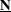 can be diagonalized as

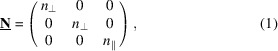

where *n*_∥_ and 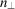 refer to the parallel and perpendicular indices of refraction, respectively. We will refer to 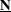 as the refractive index for brevity, with the understanding that it is actually a convenient 3D analog of the complex index of refraction.

### 3.4. Mathematical representation of P-RSoXS

The mathematical operations that mimic P-RSoXS can be divided into six steps.

(i) Effective refractive index. For each voxel, the effective refraction tensor for material component *j* can be computed using the aligned and unaligned fractions as

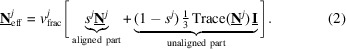




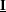
 is the identity matrix, a square matrix in which all diagonal elements are one and all off-diagonal elements are zero.

(ii) Rotated refractive index. For each material component *j* in every voxel, the effective refractive tensor 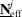 is rotated according to the alignment vector 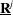 ,

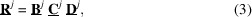

where 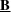 , 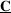 and 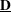 are the rotation matrices following the Euler angle convention depicted in Fig. 2 ▸. The rotated refractive index 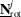 is computed as

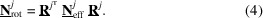




(iii) Polarization computation. The induced molecular polarization **p** produced by the electric field **e** of the beam is computed as

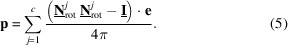

Voxel-to-voxel differences in the **p** components are the origin of scattering contrast in P-RSoXS. The structure of these components in real space can be complex, even for simple structures. For a qualitative picture of this complexity, Fig. 1 ▸ shows an illustration of *p*_*x*_ and *p*_*y*_ magnitudes for a simple compositionally homogeneous disc with radial orientation of a uniaxial dielectric function (polyethylene in this image), at an energy that enhances orientation contrast in the material. In Fig. 1 ▸, the initial morphology is shown in the bottom left. Moving right in the direction of beam passage, the absolute value of the *p*_*x*_ component is shown on the right with a pink false-color map and the absolute value of the *p*_*y*_ component is shown on the left with a green false-color map. The initial beam in this diagram is shown as polarized parallel to the *x* axis. The ‘polarized’ *p*_*x*_ components describe the field that remains polarized parallel to the *x* axis after interaction with the sample. The ‘ellipsometric’ (also called ‘depolarized’) *p*_*y*_ components describe the field that is polarized parallel to the *y* axis after interaction with the sample. Models that include Euler angle tilt relative to the *z* axis may also contain *p*_*z*_ components (not shown). The scattering from each of the *p*_*x*_ and *p*_*y*_ components is then shown, followed by their sum in the far-field projection.

We include a switch to allow the final scattering pattern to be computed by averaging across different orientations of the electric field. If this computation is enabled, it is performed as follows: We start with **e** = (1, 0, 0) and we rotate **e** using a rotation matrix 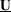 . The rotation is done in fine increments across a range and then averaged. This rotation functionality is included as a capability to smooth simulated pattern features that arise from the finite size of the models. Rotating in small increments and averaging the scattering pattern in this way effectively simulates a non-interacting polydomain material where each domain is a copy of the original model that is rotated about the *z* axis. Enabling this functionality will better capture electric field interactions with model details. This functionality should not be used, however, if a model has structural features that are intentionally non-uniform in the *xy* plane directions.

(iv) Fast Fourier transform (FFT). To get the reciprocal space (**q**) representation, we first compute the FFT of the real-space polarization vector **p**,

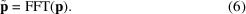




(v) Scatter computation. The differential scattering cross section *X*(**q**) is given by

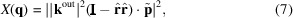

where 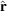 is the real-space unit vector from the sample to the detector, such that **r** ≃ **k** ^out^ = **k** ^in^ + **q**, **k** ^in^ is the wavevector of the incident wave and **k** ^out^ is the wavevector of the outgoing wave. Equation (7) is derived using the first-order Born approximation (far-field limit) (Born & Wolf, 2013 ▸). The individual components of 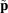 are combined to produce the final pattern simulation. Molecular orientation that gives rise to anisotropy in the real-space structure of **p** will produce correspondingly anisotropic patterns in the reciprocal-space structure of the elements of 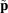 , as illustrated for the disk morphology in Fig. 1 ▸. There is typically a significant difference between the intensity of the polarized and de­polarized scattering components such that the polarized scattering contributes most strongly to the sum, but the depolarized components remain essential for accurate simulation. This sum is not shown in Fig. 1 ▸ because a final step is required, the Ewald projection.

(vi) Ewald projection. The final step consists of projecting the differential scattering cross section onto the Ewald sphere to mimic the detector. For this step we will separately consider the elements of **q** as *q*_*x*_, *q*_*y*_ and *q*_*z*_. For each location on the detector given by (*q*_*x*_, *q*_*y*_), we compute *q*_*z*_ by evaluating 

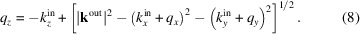

For real values of *q*_*z*_, the detector image is given by interpolating *X*(**q**). Interpolation is needed because *q*_*z*_ may not be an integer. We perform linear interpolation using the nearest integer neighbors. Fig. 1 ▸ shows the final scattering simulation after Ewald projection of the 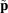 components, also depicted.

## 4. Algorithm

The two criteria considered during algorithm design for P-RSoXS simulation are the memory limitation on the GPU side and the communication time from the central processing unit (CPU) to the GPU. GPU architecture advancements have produced constant memory growth, but GPU memory remains much lower than its CPU counterpart. Additionally, data communication from CPU to GPU or *vice versa* remains a bottleneck. In this section, we describe the memory layout for the morphology and describe the two algorithms supported by our framework: (i) Algorithm 1, which minimizes the data movement from CPU to GPU but is memory intensive, especially for larger numbers of material components; and (ii) Algorithm 2, which minimizes the memory footprint at the cost of communication between the CPU and GPU.

### 4.1. Memory layout for morphology

**Figure 3:**
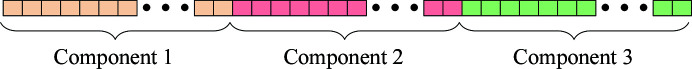
An illustration of the memory layout of morphology for a *c* = 3 component system, color coded for each component. The complete morphology is a 1D array of size *N*_*x*_ × *N*_*y*_ × *N*_*z*_ × *c*. Each entry of morphology consists of a Real4 entry.

The overall morphology is represented in memory as a 1D array of size *N*_*x*_ × *N*_*y*_ × *N*_*z*_ × *c*. Each entry of this 1D array consists of a Real4 2 data type representing the four components (*v*_frac_, *s*, φ, θ). Fig. 3 ▸ shows the memory layout of the morphology for P-RSoXS simulation. Using a 1D array ensures that only a single cudaMemcpy instruction is needed to load from CPU to GPU memory. The use of the Real4 data type ensures vectorized load from global memory of the GPU to local memory. Additionally, this memory layout – of striding through voxels first before striding through components – ensures the best utilization of the load bandwidth from global memory to local memory.

The memory layout allows for additional computational gains during the averaging process. An earlier algorithm by Gann *et al.* (2016 ▸) relied on rotating the material while keeping **e** fixed, in order to compute the average intensity on the detector. This step is computationally expensive, especially for 3D morphologies, where we would need to rotate *N*_*z*_ channels of *N*_*x*_ × *N*_*y*_ voxelated morphologies. In this work, we reformulated the algorithm to rotate **e** while keeping the material fixed.

Additionally, we rotate the detector coordinates in the last step to average the resulting intensity. The transfer of computation from the material to the **e** reference frame makes the algorithm computationally efficient and GPU friendly.

### 4.2. Communication minimization (Algorithm 1)


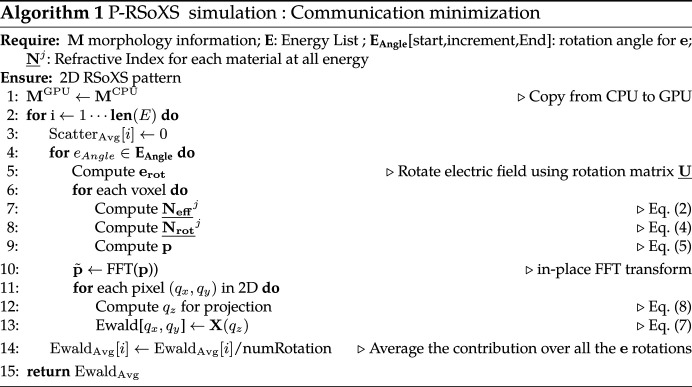


This algorithm[Chem scheme1] relies on copying all the morphology information once from the CPU to the GPU at the start of the computation, and this is then utilized for all subsequent computations. Once this copy has been performed, no further communication is needed for the next computation steps. We perform the polarization computation **p** given by equation (5). As discussed in the previous section, the memory layout for the vector morphology allows us to achieve maximum bandwidth, mainly because all subsequent threads within the block try to load the nearby memory. Additionally, packing the data as Real4 allows us to perform vectorized load from global memory to local thread memory. To utilize the available resources efficiently, we use streams to compute the FFT of the polarization vector. In particular, we use three streams, one for each of *p*_*x*_, *p*_*y*_ and *p*_*z*_. We then compute the *q*_*z*_ position for a given value of (*q*_*x*_, *q*_*y*_) 2D pixel, given by equation (8). Note that we only compute *X*(*q*_*z*_) for the pixels participating in the 3D Ewald projection. This helps to eliminate the memory requirement to store a 3D vector for *X*(**q**). Finally, the averaged result (averaged across a range of rotation angles of **e**) is transferred from the GPU to the CPU. Table 1 ▸ shows the memory requirement for P-RSoXS simulation.

One potential drawback of this approach is the overall memory requirement. We can see that the overall memory requirement grows linearly with the number of materials. Memory requirements are dependent on the resolution and number of materials per model and can range from less than 1 GB to approaching or exceeding the ∼ 48 GB memory limit of current-generation CUDA GPUs.

### 4.3. Memory minimization (Algorithm 2)


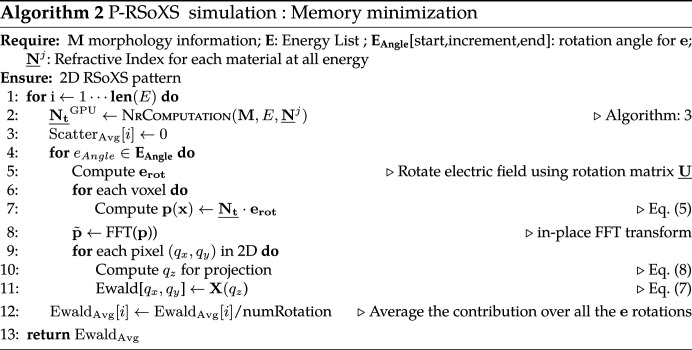



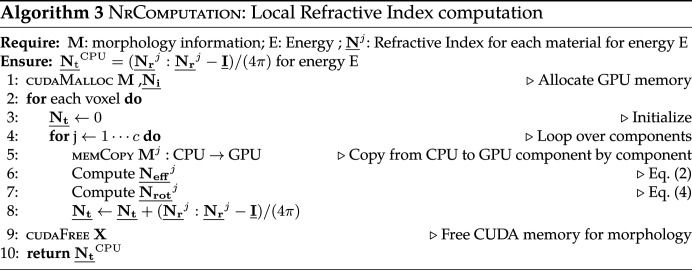


Analysis of the steps detailed in Section 3.4 indicates that morphology inputs are only required during the computation of polarization **p** in equation (5). The main idea of Algorithm 2 is to precompute a precursor of **p** for a given energy and use it for all subsequent computations (across multiple rotations of **e**). The pre-computation stage is shown by Algorithm 3, which computes an intermediate tensor 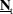 [which is defined as 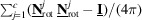 , see equation (5)]. The computation in this step is embarrassingly parallel and can be computed per voxel independently. Therefore, even if the complete memory required does not fit on the GPU, we can asynchronously stream the required data to and from the CPU and GPU. In particular, we stream the data per material from CPU to GPU. The memory requirement during this stage thus drops from (4*c* + 12)(*n*_*x*_ *n*_*y*_ *n*_*z*_) to 16(*n*_*x*_ *n*_*y*_ *n*_*z*_). The streaming helps to overlap computation with communication and hides the latency.

Once all 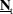 have been computed, these values are subsequently used for the P-RSoXS simulation in a similar way as in Algorithm 1. Table 2 ▸ shows the memory requirement for the different steps. The memory requirement for the main stage is independent of the number of materials and requires less memory than Algorithm 1 for *c* ≥ 3. This is an important consideration, especially when we consider multi-component chemical systems. Finally, we exploit the symmetric structure of 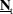 to minimize further the number of computations required. While 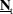 contains nine entries (3 × 3 matrix), only six of these entries are unique.

**Remark 1**

We note that further optimization is possible in terms of memory requirement. Theoretically only 6(*n*_*x*_ *n*_*y*_ *n*_*z*_) + 2(*n*_*x*_ *n*_*y*_) (three **p** vectors, and two vectors for Ewald and Ewald_Avg_) units of memory are required for P-RSoXS computation. All the other information can be communicated from CPU to GPU in a streamed fashion. But achieving this theoretical bound would imply a lot of communication overhead with **M** or 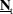 being communicated from CPU to GPU for each rotation of the **e** field. For most of our use cases, we find that the memory available on current GPUs, like the NVIDIA Voltas V100, is sufficient for carrying out the computation using Algorithm 1, with Algorithm 2 needed in some extreme cases.

**Remark 2**

We remind the reader that *c* denotes the total number of materials. While we recommend adding vacuum as an additional component to the morphology to ensure robust morphology checks, this is not strictly enforced. *CyRSoXS* does not provide any special treatment to vacuum. When provided in the input morphology, the code treats vacuum as an additional material.

## 5. Results: validation cases

We comprehensively verify and validate *CyRSoXS* by comparing against an array of benchmarks. This includes three test cases with analytical scattering expressions and one validation case consisting of comparisons against results within an earlier framework (Gann *et al.*, 2016 ▸).

### 5.1. Form factor scattering test

A simple validation case is for form factor scattering, in which scattering results purely from the shape of a particle. We specifically test the form factor scattering of a sphere. We consider two cases, a 2D projection of a sphere and a 3D sphere, and we compare the results of *CyRSoXS* with analytical expression results. The analytical expression for form factor scattering of a sphere is given by

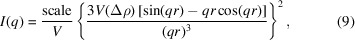

where scale is the intensity scaling, *V* is the volume of the sphere, *r* is the sphere radius (in ångströms) and Δρ is the scattering contrast (in Å^−2^).

#### 5.1.1. Two-dimensional projection of a sphere

**Figure 4:**
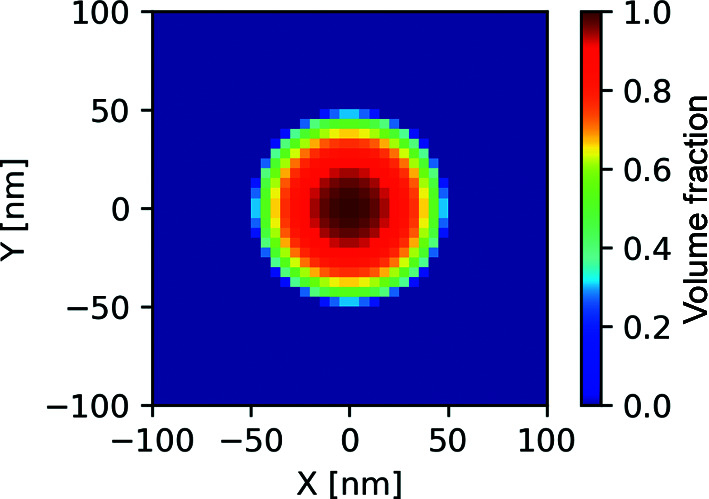
An enlarged view of the 2D projected sphere.

As a first test case, we consider a 2D projection of a sphere of radius 50 nm placed at the center of the domain. For this test, the sphere is composed of amorphous polyethylene in a surrounding medium of vacuum. Fig. 4 ▸ illustrates an enlarged view of the domain setup for the test case. The whole domain is discretized using 2048 × 2048 × 1 voxels, with each voxel representing a 5 × 5 × 5 nm physical volume. Fig. 4 ▸ shows an enlarged view near the center of the circle. The scattering profile for this morphology was simulated from 270 to 310 eV using tabulated optical constants of polyethylene (Gann, 2022 ▸) for the projected sphere and vacuum outside.

**Figure 5:**
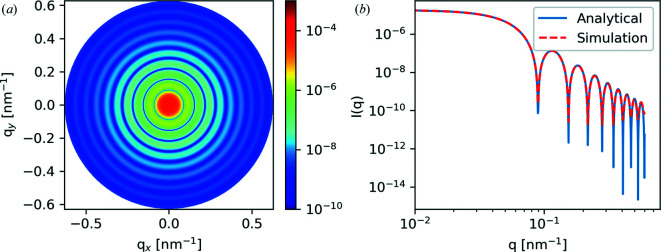
(*a*) Results of the projected sphere case and (*b*) comparison with the analytical solution.

**Figure 6:**
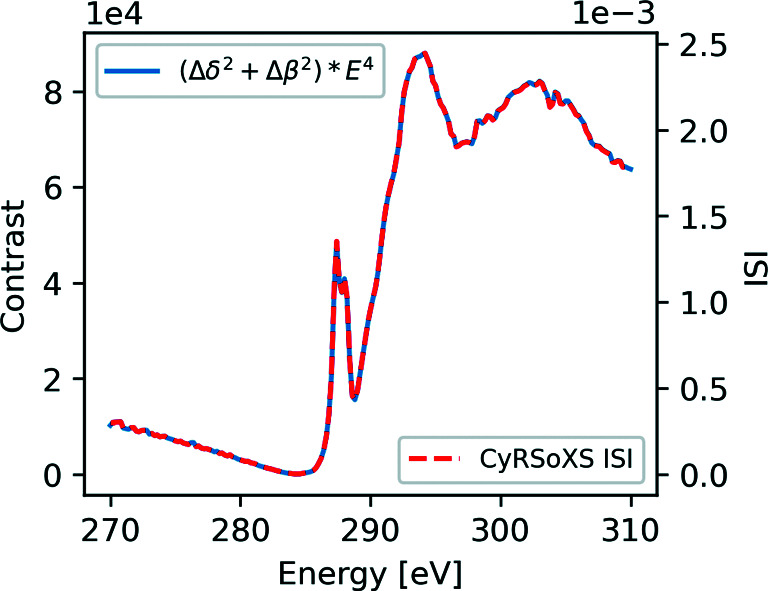
The simulated ISI alongside the theoretical energy dependence.

Fig. 5 ▸ shows the result of the 2D projected sphere validation case at 285 eV. Line cuts of the analytical and simulated data are plotted in Fig. 5 ▸(*b*) and show excellent agreement. To validate the energy dependence of the P-RSoXS simulation, we calculate the integrated scattering intensity (ISI), a *q*-bounded approximation of the scattering invariant, across the range of simulated photon energy values. Fig. 6 ▸ plots the simulated ISI alongside the theoretical energy dependence given by the analytical expressions provided by Tatchev (2010 ▸), computed for this specific dielectric function by us,

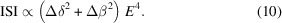

While they are on different absolute scales, the theoretical and simulated photon energy dependences show commensurate relative scaling, indicating that we are capturing the correct physics in our scattering model.

#### 5.1.2. Three-dimensional sphere test

**Figure 7:**
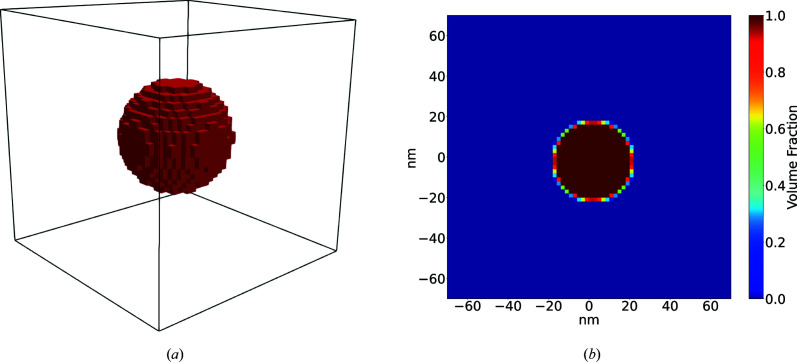
(*a*) The domain for the 3D sphere validation test (not to scale). (*b*) A 2D slice along the mid plane.

Fig. 7 ▸ shows the 3D sphere test domain along with a 2D slice of the sphere mid-plane. The morphology consists of 128 × 2048 × 2048 voxels, where each voxel is 5 × 5 × 5 nm. A 3D sphere of radius 50 nm is placed at the center. The simulation was carried out at 285 eV, using tabulated polyethylene optical constants for the sphere and vacuum for the surrounding matrix.

**Figure 8:**
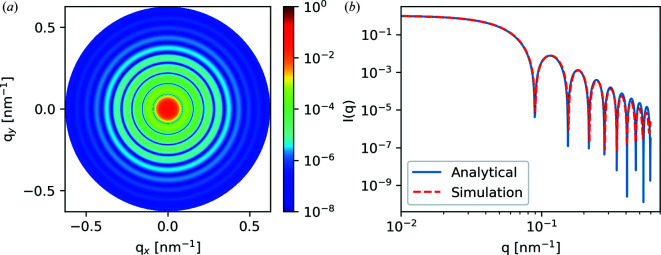
(*a*) Results of the 3D sphere case and (*b*) comparison with the analytical solution.

**Figure 9:**
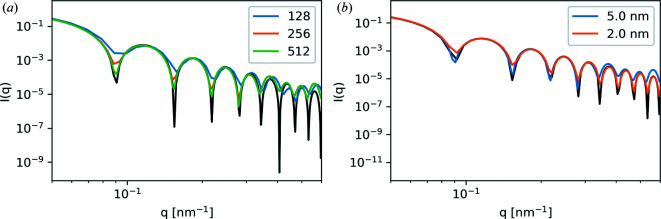
Effect of (*a*) box size and (*b*) voxel size on the 3D sphere form factor for different voxel sizes of 128^3^, 256^3^ and 512^3^ and different physical sizes of 5 and 2 nm. The black lines show the analytical results.

Fig. 8 ▸(*a*) shows the 2D scattering pattern and Fig. 8 ▸(*b*) compares the 1D analytical expression for a sphere with the azimuthally integrated data from Fig. 8 ▸(*a*). The analytical and simulation data were both normalized to 1 at *q* = 1 × 10^−2^ nm^−1^. We see an excellent comparison between the analytical and simulated results. The minor discrepancy between the simulation and analytical results at higher *q* values can be attributed to the finite discretization of the sphere and voxel size. To demonstrate this further, we simulated two additional parameter sweeps: increasing box size at a constant voxel size of 5 × 5 × 5 nm, and a constant 256 × 256 × 256 voxels at 5 and 2 nm voxel sizes. These results are plotted in Fig. 9 ▸. Increasing the box size at a constant voxel size effectively pads the sphere morphology with additional vacuum. This has the effect of creating more complete destructive interference in the form factor minima. Decreasing the voxel size at a constant box size increases the resolution of the simulation and leads to better agreement at higher *q*, but more of the simulation box is occupied by the sphere. Thus the padding is decreased and less complete destructive interference results in the minima.

### 5.2. Periodic structure test

Extending beyond form factor scattering, many materials studied with X-ray scattering techniques exhibit periodic structures which result in Bragg diffraction: constructive interference of the scattered X-rays produces sharp peaks at locations corresponding to the periodic spacing. Materials of this nature that have been studied with RSoXS include block copolymers (Wang *et al.*, 2011 ▸; Virgili *et al.*, 2007 ▸) and patterned thin films (Freychet *et al.*, 2018 ▸). Voxel­ized representations will approximate the spacings and shapes of real morphologies. We perform two validation cases that reflect this periodic arrangement of structures, a 2D hexagonal packed lattice and a grating test.

#### 5.2.1. Circle on hexagonal lattice

**Figure 10:**
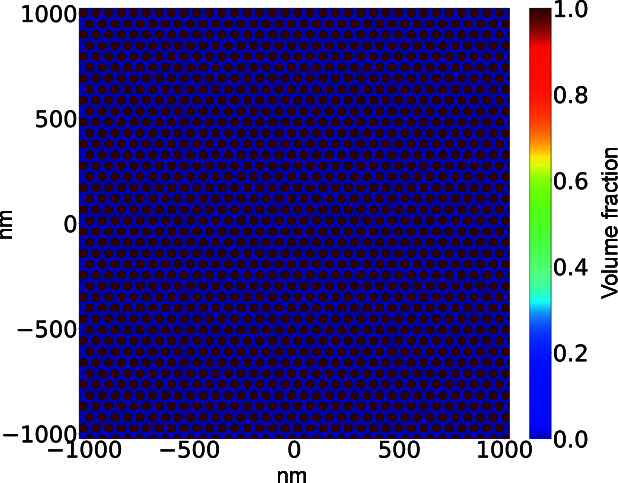
A volume fraction map of PEOlig for the hexagonal lattice. The dark-blue region represents vacuum.

We first consider an arrangement of circular domains on a 2D hexagonal lattice (Fig. 10 ▸). This morphology is representative of hexagonally packed cylinders, a common block-copolymer morphology. We consider the cylinders to be oriented parallel to the X-ray beam.

**Figure 11:**
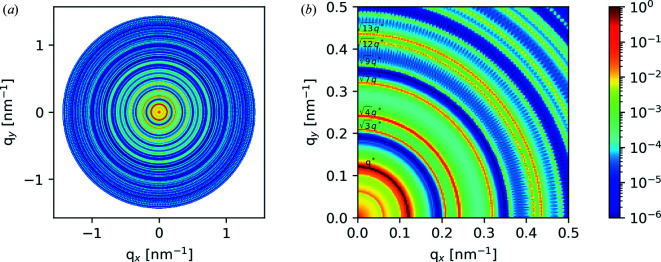
(*a*) The hexagonal lattice validation case. (*b*) Contours of *I*(**q**) with the corresponding peak locations.

**Figure 12:**
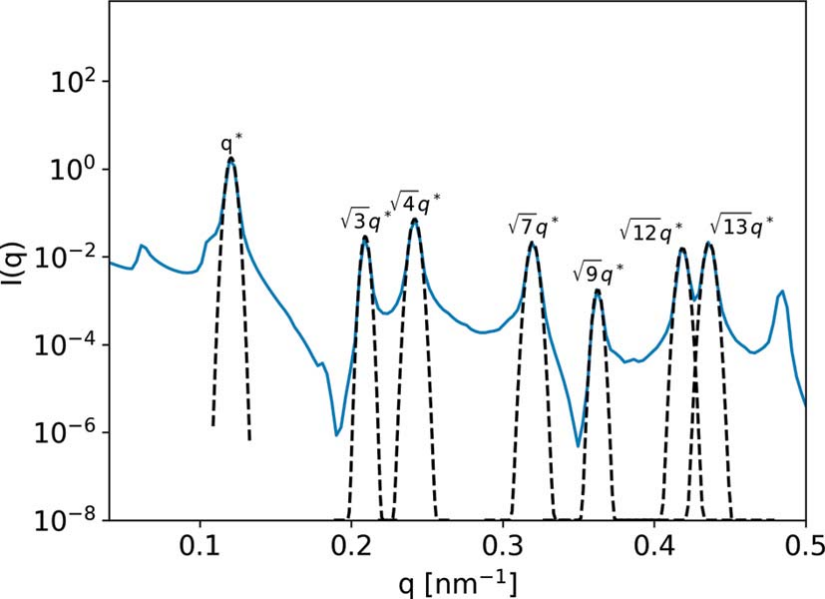
A 1D simulated diffraction pattern (solid blue line) with the analytical peak locations marked (dashed lines).

Fig. 11 ▸ shows the 2D scattering pattern output from *CyRSoXS*. Given the target lattice spacing of the input morphology, we observe Bragg peaks at the expected locations [q*, (3^1/2^)q*, (4^1/2^)q*, (7^1/2^)q*, (9^1/2^)q* and so on]. Fig. 12 ▸ shows the azimuthally integrated scattering intensity plotted versus *q*, with the first seven Bragg peaks labeled. There is perfect agreement between the analytical and simulated peak locations. We do observe some non-peak background features with low intensity that originate from the finite size of the model and voxel-level discretization effects. Such artifacts can be further reduced by using larger and/or higher-resolution models, models that contain realistic structural defects, and models with periodic boundary conditions.

#### 5.2.2. Grating test

**Figure 13:**
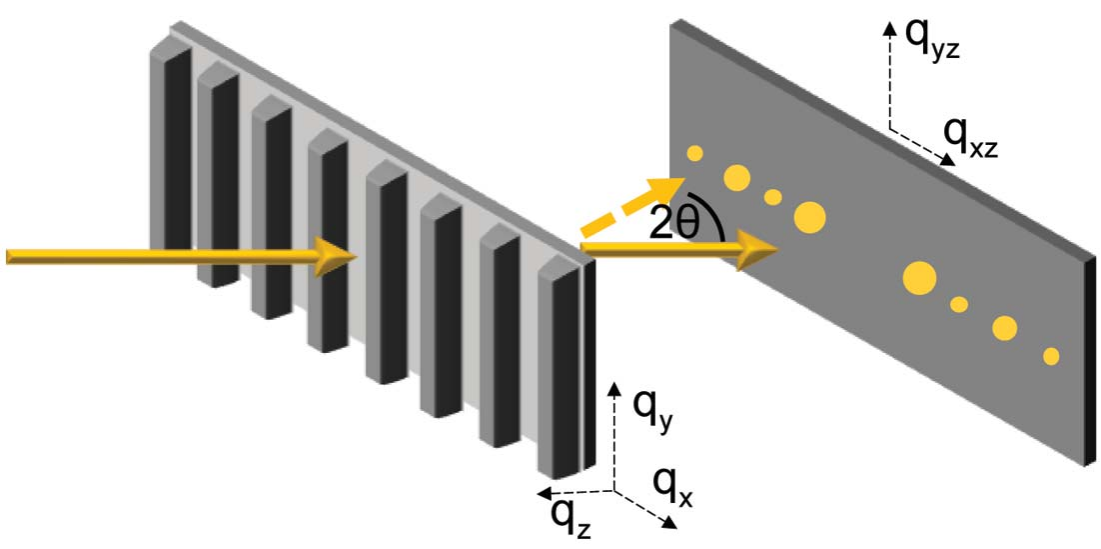
The setup of the line-grating simulation.

**Figure 14:**
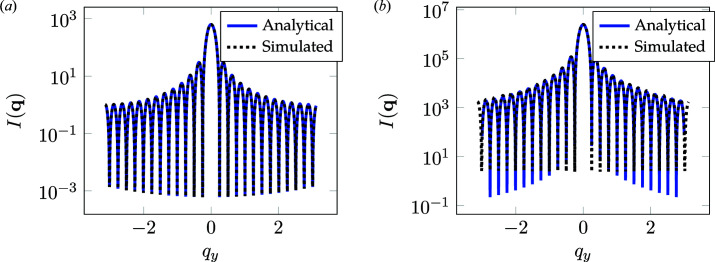
Comparisons of analytical and simulation line-cut integration for (*a*) 2D and (*b*) 3D line gratings. The *q*_*x*_ component of **q** is at the location of the first-order peak.

The second periodic structure test case is a set of parallel lines which form a grating structure. This type of morphology is observed in the directed self-assembly of block copolymers or in structures fabricated using lithographic processes; it is often seen in semiconducting manufacturing. We consider a single line grating morphology in two and three dimensions. The 3D morphology consists of a single line grating extended in the *z* direction. Fig. 13 ▸ shows the setup for the grating simulation. The 2D morphology consists of 1024 × 1024 × 1 voxels whereas the 3D morphology consists of 1024 × 1024 × 63 voxels, with each voxel representing a physical dimension of 1 × 1 × 1 nm. The simulation was carried out at 17 keV. The analytical results are calculated using a previously published procedure (Sunday *et al.*, 2015 ▸) in which the grating is discretized into a stack of trapezoids. The analytical solution for the Fourier transform of a trapezoid is used to calculate the scattering intensity at each *q* position. Fig. 14 ▸ compares the analytical and simulation results for the line gratings. The simulated results are in excellent agreement with the analytical results.

### 5.3. Orientation effect on polymer-grafted nanoparticles

**Figure 15:**
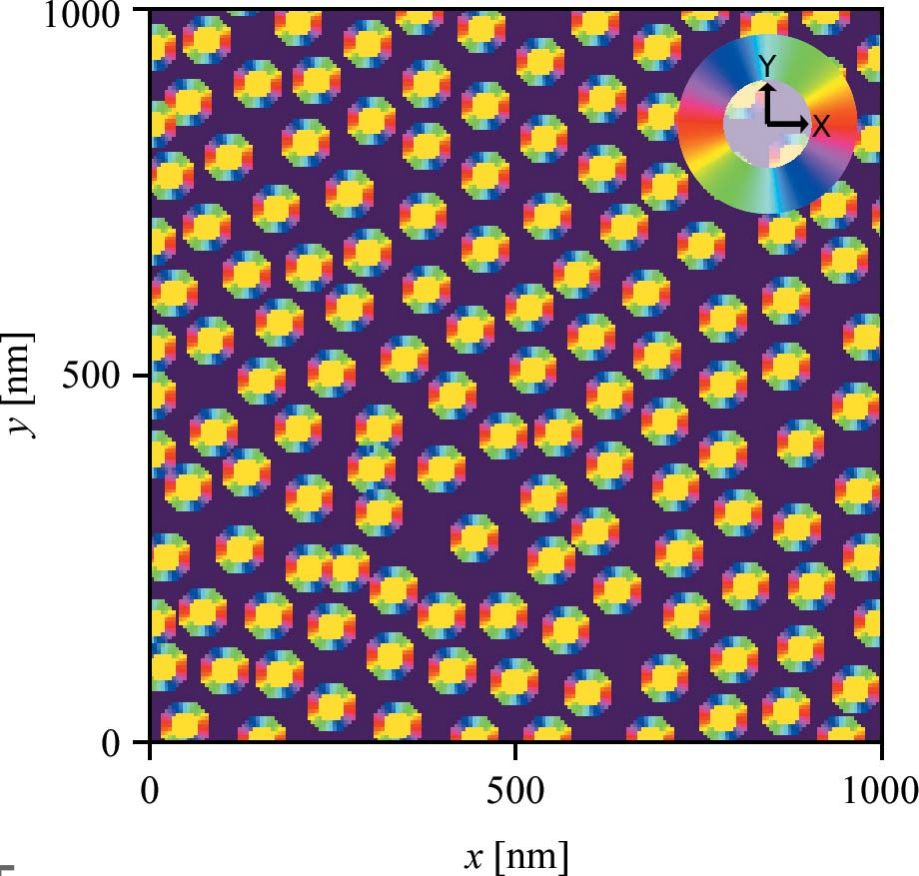
A 2D slice of 3D PGN morphology. An oriented shell of polystyrene (PS) surrounds each gold nanoparticle core. The pixels in this image are colored by the values of the Euler angle φ^PS^, which exhibits a radial orientation relative to the particle centers. The orientation of the extraordinary axis of the dielectric function in real space relative to the *x* and *y* axes is shown in the inset color wheel. This 2D slice was collected near the particle equators such that φ^PS^ ≃ π/2 for all pixels.

**Figure 16:**
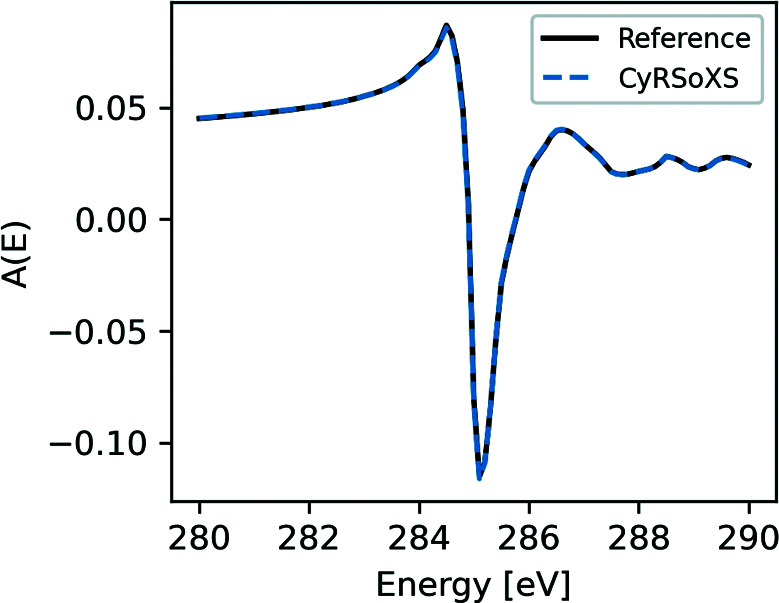
Scattering anisotropy plotted versus energy for the *CyRSoXS* and reference simulators

All of the previous test cases deal with isotropic materials. As the final validation case, we consider a film of polymer-grafted nanoparticles (PGNs) (Mukherjee *et al.*, 2021 ▸). Polystyrene chains are grafted onto gold nanoparticles and the confinement of polystyrene chains near the nanoparticle surface results in radial stretching of the chains and a net molecular orientation. Fig. 15 ▸ is a 2D slice of the 3D morphology, showing the gold nanoparticle core surrounded by the oriented polystyrene shell, all embedded in a matrix of isotropic polystyrene. The *CyRSoXS* simulation is tested against the current state-of-the-art P-RSoXS simulator (Gann *et al.*, 2016 ▸). Fig. 16 ▸ plots the scattering anisotropy averaged over *q* = 0.02–0.4 nm^−1^ for the reference simulator and for our GPU-accelerated P-RSoXS simulator. Our implementation perfectly reproduces the results of the reference simulator.

## 6. Performance

In this section, we report the scaling of *CyRSoXS* with respect to variation in the numbers of voxels and materials. All computations were carried out using an NVIDIA Volta V100 GPU with 32 GB of memory.

### 6.1. Performance with increasing number of voxels

As our first scaling test, we considered performance with increasing number of voxels. The overall number of voxels varied from 128 × 128 × 16 to 1024 × 1024 × 128 with an increment of 2× in each direction (the 1024 × 1024 × 128 voxel size is the largest that fits into the memory of a 32 GB NVIDIA V100 GPU). The number of materials is fixed to four and the computation was carried out for 150 photon energies. For each photon energy, the electric field **e** was rotated from 0 to 180° in increments of 2°.

**Figure 17:**
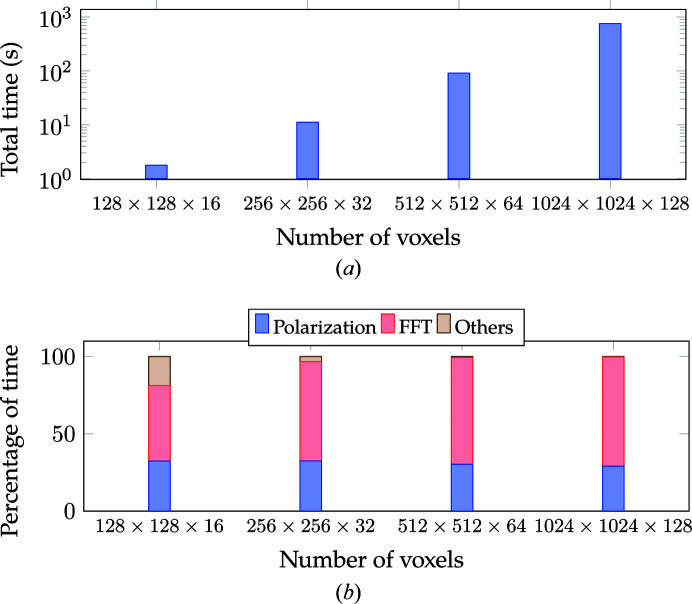
The performance of Algorithm 1 with variation in the number of voxels. (*a*) Total time with variation in the number of voxels. (*b*) Percentage of time with variation in the number of voxels. The number of materials was fixed to four. The time reported corresponds to computation of 150 energy levels with **e** rotated from 0 to 180° in increments of 2° for each energy level.

Fig. 17 ▸ compares the time with increasing number of voxels for Algorithm 1. Fig. 17 ▸(*a*) shows the variation in total wall time with respect to the number of voxels. Overall we see a linear dependence 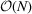 , where *N* is the total number of voxels. Fig. 17 ▸(*b*) compares the percentage of time taken by different sections of the computation. The total time is dominated by polarization computation [equation (5)] and FFT computation. The ‘Others’ cost, which includes Ewald projection computation, image rotation, and data transfer from CPU to GPU and *vice versa*, forms a significant fraction at lower resolution (*i.e.* smaller voxel sizes) but becomes insignificant at higher resolutions.

**Figure 18:**
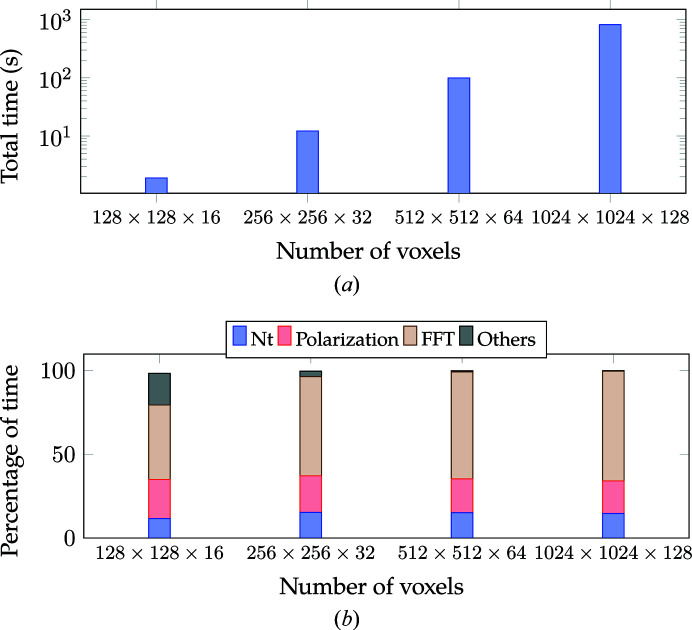
The performance of Algorithm 2 with variation in the number of voxels. (*a*) Total time with variation in the number of voxels. (*b*) Percentage of time with variation in the number of voxels. The number of materials was fixed to four. The time reported corresponds to computation of 150 energy levels with **e** rotated from 0 to 180° in increments of 2° for each energy level.

Fig. 18 ▸ compares the time with increasing number of voxels for Algorithm 2. Fig. 18 ▸(*a*) shows the variation in total time whereas Fig. 18 ▸(*b*) compares the percentage of time with increasing number of voxels. We observe a similar performance behavior to Algorithm 1, including 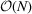 scaling with increasing number of voxels. The majority of the time is spent in computing 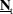 , which also involves copying data from the CPU to the GPU (Algorithm 3), polarization computation and FFT computation. The ‘Others’ cost, similarly to the previous algorithm, forms a significant percentage at lower resolution but becomes insignificant at higher resolutions.

### 6.2. Performance with increasing number of materials: communication minimization versus memory minimization algorithms

In our next analysis, we compared the performance of both algorithms with respect to increasing number of materials. We considered a system with a voxel size of 2048 × 2048 × 64. The computation was carried out for nine photon energies. For each photon energy, the electric field **e** was rotated from 0 to 180° in increments of 2°. We utilized ten streams for the computation of Algorithm 2 to overlap computation and communication.

**Figure 19:**
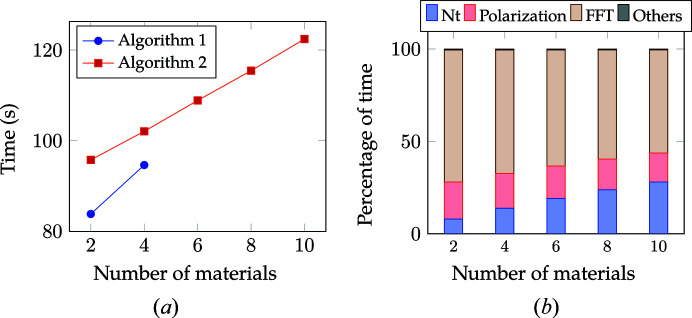
Run time and percentage distribution of P-RSoXS for 2048 × 2048 × 64 morphology with increasing number of materials for nine different energy levels. (*a*) Run time. (*b*) Percentage of time for Algorithm 2. The parameter **e** was rotated from 0 to 180° in increments of 2° for each energy level. For Algorithm 1, the overall memory requirement exceeds the GPU memory size if the number of materials is greater than four.

Fig. 19 ▸(*a*) compares the total time for the two algorithms. Algorithm 1 is faster than Algorithm 2. However, the overall slope, or the rate of increase in time with number of materials, tends to be much steeper for Algorithm 1 than Algorithm 2. This is because the polarization computation [equation (5)] involves a loop over the number of materials. Algorithm 1 performs this computation for each rotation of **e**, whereas in the case of Algorithm 2 this computation is carried out once for each photon energy and stored in 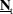 . With increasing number of materials, this computation tends to dominate and thus we see a higher slope for Algorithm 1. Further, we observe that the memory requirement of Algorithm 1 exceeds the overall GPU memory for more than four materials, whereas Algorithm 2 continues to exhibit a linear variation with increasing number of materials. This agrees with the memory requirement analysis in Section 4. We recall that the memory requirement of Algorithm 1 exceeds that of Algorithm 2 for number of materials ≥ 3.

Fig. 19 ▸(*b*) shows the percentage of time for different sections of Algorithm 2. We see an increase in time for 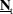 computation. This is expected, as only 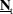 computation in Algorithm 2 depends on the number of materials. Overall, the time is mostly dominated by FFT computations.

### 6.3. Scaling performance across multiple GPUs

We parallelize the code with respect to photon energies across multiple GPUs. This makes the code embarrassingly parallel. Each GPU device allocates its own chunk of memory depending on the photon energies owned by it and performs the computation independently. We utilize *OpenMP* to schedule the threads, with each thread handling a single GPU. This allows us to utilize all GPUs efficiently across a single node.

**Figure 20:**
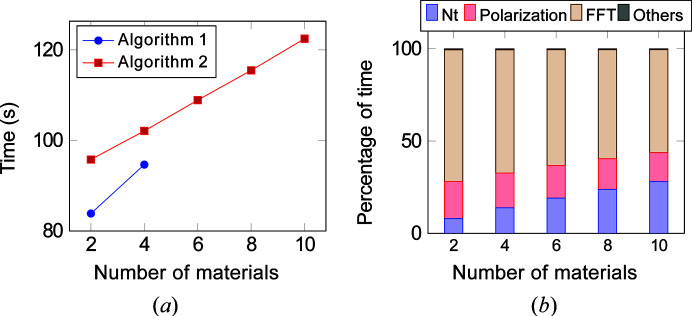
Scaling of the P-RSoXS simulator on multiple GPUs. (*a*) Scaling for 512 × 512 × 64 voxels. (*b*) Scaling for 1024 × 1024 × 128 voxels. The number of materials was fixed to four. The time reported corresponds to computation of 150 energy levels distributed across multiple GPUs with **e** rotated from 0 to 180° in increments of 2° for each energy level.

In order to demonstrate the scaling performance, we consider a server with two NVIDIA V100 GPUs and analyze the efficiency for different voxel sizes (Fig. 20 ▸). We consider a material system with two different voxel sizes of 512 × 512 × 64 and 1024 × 1024 × 128 and four materials. We consider 150 photon energies distributed across multiple GPUs. Overall, we see an ideal scaling behavior, with both algorithms achieving 2× acceleration while utilizing two GPUs.

**Remark 3**

In practice, we observe Algorithm 1 to be faster than Algorithm 2. Therefore, Algorithm 1 is recommended as the first choice, until we hit the memory limit of the GPU (usually exhibited as a memory error).

### 6.4. Comparison with current state of the art

We consider the PGN case from Section 5.3 for performance comparison between *CyRSoXS* and an *Igor*-based state-of-the-art simulation (Gann *et al.*, 2016 ▸). The overall morphology contains 512 × 512 × 32 voxels with three components. We only report the timing for one rotation and 101 photon energies. The *Igor*-based simulation took around 31 min on an Intel Core i7-8700 CPU running at 3.20 GHz with 24.0 GB of RAM. In contrast, *CyRSoXS* took only 1.05 s (>1000× acceleration) on an NVIDIA Quadro A6000 GPU with 48 GB of GDDR6 global memory to accomplish this task. We note that the acceleration will become much more prominent once we perform the simulation for multiple rotations, as these rotations do not involve any communication between the CPU and GPU.

## 7. Python interface to *CyRSoXS*

In addition to GPU acceleration, we have added a Python interface using *Pybind11* (https://github.com/pybind/pybind11). *Pybind11* was designed to expose C++ data types to Python and *vice versa*. One of the benefits of this approach is directly passing the morphology information via memory instead of performing file I/O operations, which can be a major bottleneck for fitting and other inverse problems. Additionally, the output of the scattering pattern in the form of *NumPy* arrays enables users to use sophisticated Python visualization libraries like *matplotlib* (Barrett *et al.*, 2005 ▸) and *seaborn* (Waskom, 2021 ▸) and to develop Python-based post-processing tools. We also interface with the *cupy* (Nishino & Loomis, 2017 ▸) library that enables morphology generation on a GPU. A morphology generated on a GPU can be directly passed to the simulator without copying data back and forth from the CPU. However, the morphology layout must strictly match the framework layout as shown in Fig. 3 ▸ and described in Section 4.1.

We believe that the availability of the Python interface will give a major boost to inverse problems relating to materials design, as most of the machine learning (ML) or data analysis (DA) toolkits (Garreta & Moncecchi, 2013 ▸; Chollet, 2015 ▸; Abadi *et al.*, 2016 ▸; Paszke *et al.*, 2019 ▸) are currently Python based. This interface will allow users to integrate their ML/DA models seamlessly with the current framework.

## 8. Conclusions

We have demonstrated a new P-RSoXS virtual instrument with greatly increased performance compared with the current state of the art. Computations with this new virtual instrument are fast enough to enable practical data fitting by adjusting structure parameters using goal-seeking algorithms.

The first fitting of orientational parameters to experimental P-RSoXS data was recently demonstrated using this virtual instrument to simulate polymer-grafted nanoparticles using a high-throughput multi-resolution parametric sweep of a three-parameter system (Mukherjee *et al.*, 2021 ▸).

We have developed Python-driven workflows that demonstrate the practical use of this virtual instrument with other fitting methods, including genetic algorithm and Markov-chain Monte Carlo approaches, and these will be published elsewhere. Close integration with Python environments affords opportunities to develop morphological models based on data fusion approaches, particularly exploiting real-space imaging, which reduces common questions of model uniqueness in fitting small-angle scattering data.

The P-RSoXS virtual instrument shows great promise as a cornerstone of future approaches for assimilating complementary data streams to construct complex and self-consistent material structure representations *in silico*, and ultimately for powering inverse design frameworks that eliminate the need for costly Edisonian optimization approaches.

## 9. Data and software availability

The core C++/CUDA software is available online at https://github.com/usnistgov/cyrsoxs. Additional reference data and analysis scripts necessary to reproduce the validation results in Section 5 are available online at https://github.com/usnistgov/NRSS under tests/validation.

## Figures and Tables

**Table 1:** Memory requirement for various steps during P-RSoXS computation for Algorithm 1

Algorithm	Variable	Data type	Size	Total size
P-RSoXS (Algorithm 1)	**M**	Real	4*c*(*n*_*x*_ *n*_*y*_ *n*_*z*_)	4*c*(*n*_*x*_ *n*_*y*_ *n*_*z*_)
	*p*_*x*_	Complex	(*n*_*x*_ *n*_*y*_ *n*_*z*_)	2(*n*_*x*_ *n*_*y*_ *n*_*z*_)
	*p*_*y*_	Complex	(*n*_*x*_ *n*_*y*_ *n*_*z*_)	2(*n*_*x*_ *n*_*y*_ *n*_*z*_)
	*p*_*z*_	Complex	(*n*_*x*_ *n*_*y*_ *n*_*z*_)	2(*n*_*x*_ *n*_*y*_ *n*_*z*_)
	Ewald	Real	(*n*_*x*_ *n*_*y*_)	(*n*_*x*_ *n*_*y*_)
	Ewald_Avg_	Real	(*n*_*x*_ *n*_*y*_)	(*n*_*x*_ *n*_*y*_)
	Total	(4*c* + 6)(*n*_*x*_ *n*_*y*_ *n*_*z*_) + 2(*n*_*x*_ *n*_*y*_)		

**Table 2:** Memory requirement for various phases during P-RSoXS computation for Algorithm 2 and for NrComputation (Algorithm 3)

Algorithm	Variable	Data type	Size	Total size
P-RSoXS (Algorithm 2)	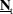	Complex	6(*n*_*x*_ *n*_*y*_ *n*_*z*_)	12(*n*_*x*_ *n*_*y*_ *n*_*z*_)
	*p*_*x*_	Complex	(*n*_*x*_ *n*_*y*_ *n*_*z*_)	2(*n*_*x*_ *n*_*y*_ *n*_*z*_)
	*p*_*y*_	Complex	(*n*_*x*_ *n*_*y*_ *n*_*z*_)	2(*n*_*x*_ *n*_*y*_ *n*_*z*_)
	*p*_*z*_	Complex	(*n*_*x*_ *n*_*y*_ *n*_*z*_)	2(*n*_*x*_ *n*_*y*_ *n*_*z*_)
	Ewald	Real	(*n*_*x*_ *n*_*y*_)	(*n*_*x*_ *n*_*y*_)
	Ewald_Avg_	Real	(*n*_*x*_ *n*_*y*_)	(*n*_*x*_ *n*_*y*_)
	Total	18(*n*_*x*_ *n*_*y*_ *n*_*z*_) + 2(*n*_*x*_ *n*_*y*_)		
				
NrComputation (Algorithm 3)	**M**	Real	4*c*(*n*_*x*_ *n*_*y*_ *n*_*z*_)	4*c*(*n*_*x*_ *n*_*y*_ *n*_*z*_)
	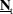	Complex	6(*n*_*x*_ *n*_*y*_ *n*_*z*_)	12(*n*_*x*_ *n*_*y*_ *n*_*z*_)
	Total (non-stream)	(4*c* + 12)(*n*_*x*_ *n*_*y*_ *n*_*z*_)		
	Total (stream)	(4 + 12)(*n*_*x*_ *n*_*y*_ *n*_*z*_)		

## Footnotes

1 Certain commercial equipment, instruments or materials are identified in this paper in order to specify the experimental procedure adequately. Such identification is not intended to imply recommendation or endorsement by NIST, nor is it intended to imply that the materials or equipment identified are necessarily the best available for the purpose.

2
Real4 represents float4, a single-precision floating-point number, or double4, a double-precision floating-point number, depending upon the type of compilation.
